# Neighbourhood deprivation and diet quality among a nationally representative sample of adults in Canada: an intersectional investigation

**DOI:** 10.1017/S1368980026103218

**Published:** 2026-07-20

**Authors:** Natalie Doan, Dana Lee Olstad, Michael Wallace, Elena Neiterman, Martin Cooke

**Affiliations:** 1 School of Public Health Sciences, University of Waterloo, Canada; 2 Department of Community Health Sciences, University of Calgary, Canada; 3 Department of Statistics and Actuarial Science, University of Waterloo, Canada; 4 Department of Sociology and Legal Studies/School of Public Health Sciences, University of Waterloo, Canada

**Keywords:** Neighbourhood deprivation, Diet quality, Health equity, Intersectionality, Public health

## Abstract

**Objective:**

The environments where individuals live can shape their dietary patterns, but research using intersectionality as a context-informed analytic tool to assess associations between neighbourhood deprivation and diet quality is lacking. We therefore examined whether four dimensions of neighbourhood deprivation were independently and/or jointly associated with diet quality among adults in Canada.

**Design:**

We used 24-h dietary recall data to calculate Healthy Eating Index (HEI-2015) scores to assess diet quality and the 2016 Canadian Marginalisation Index (CAN-Marg) to assess four dimensions of neighbourhood deprivation (material resources, immigration/visible minority, age/labour force and households/dwellings). Weighted linear regression models were used to examine whether these CAN-Marg dimensions were independently and/or jointly associated with diet quality, adjusting for individual, household and area characteristics.

**Setting:**

The ten Canadian provinces.

**Participants:**

Adults (≥ 18 years) who participated in the 2015 Canadian Community Health Survey-Nutrition.

**Results:**

The material resources (*β* = –0·955, 95 % CI = –1·438, –0·471) and immigration/visible minority (*β* = 0·768, 95 % CI = 0·268, 1·268) dimensions were independently associated with HEI-2015 scores. Significant interactions suggest the associations between living in a neighbourhood with less material resources and lower HEI-2015 scores were stronger in areas with a smaller proportion of recent immigrants/visible minorities (*β* = 0·521, 95 % CI = 0·174, 0·868) and non-working individuals (*β* = –0·643, 95 % CI = –1·052, –0·234).

**Conclusion:**

Neighbourhood material deprivation was not uniformly associated with poor diet quality. This suggests that associations between neighbourhood deprivation and diet quality are not universal and vary across neighbourhoods with differing immigrant, visible minority, age and labour force compositions.

Poor diet quality, based on conformity with dietary recommendations^(1)^, increases the risk of morbidity and mortality^(2,3)^. The quality of individuals’ dietary patterns is often defined in relation to conformity with dietary recommendations^(4)^ including guidance on adequacy, moderation, variety and balance of nutrient and food intake^(5)^. Researchers have developed and validated summary indices of overall diet quality that combine two or more components of healthy dietary patterns on the same scale^(1)^, such as the Healthy Eating Index (HEI-2015), which assesses dietary adequacy and moderation^(6,7)^.

Determinants of diet quality are multilevel and can include individual, household and neighbourhood factors^(8)^. At the individual and household levels, differences in diet quality have been observed by dimensions of socio-economic position, including sex/gender, race/ethnicity, education, income, household composition and food insecurity status^(9–12)^. At the neighbourhood level, many studies have found that neighbourhood food and socio-economic environments are associated with dietary intake^(13–21)^, in particular, diet quality^(19–21)^.

Internationally, studies have found that neighbourhood deprivation is associated with dietary intake^(20,22)^. For example, in a study among adults in seven countries, higher neighbourhood deprivation was associated with lower fruit intake in Canada, New Zealand and Scotland^(22)^. In Canada, Murphy *et al.* (2022) found that individuals living in neighbourhoods with greater material and social deprivation were less likely to meet recommendations for fruit and vegetable intake^(19)^. Furthermore, Gilham *et al.* (2020) found that diet quality was lower in the most, compared to the least, socially deprived neighbourhoods in Canada^(20)^. However, there are two key gaps in the literature pertaining to associations between neighbourhood deprivation and diet quality.

First, most studies that have examined neighbourhood deprivation in relation to dietary outcomes have assessed associations between neighbourhood deprivation and intake of specific food groups, such as fruits, vegetables or fast food^(22)^, which do not capture the quality of overall dietary intake^(1)^. Diet quality indices, such as the Healthy Eating Index (HEI-2015)^(6)^, provide insight into overall diet quality because it assesses multiple aspects of healthy eating on the same scale^(1)^. Second, researchers have not taken an intersectional approach^(23)^ that considers how aspects of neighbourhood deprivation might interact to shape individuals’ diet quality. While intersectional frameworks have often been used to describe intersecting social identities/positions at the individual level, the places where individuals live also have multiple dimensions that might interact to shape individual’s dietary patterns^(24)^.

Given the limited knowledge about how dimensions of neighbourhood deprivation may interact to affect diet quality, we examined whether four dimensions of neighbourhood deprivation were independently and/or jointly associated with diet quality among a nationally representative sample of adults in Canada using intersectionality as a context-informed tool^(24)^ to guide our analyses of various socioeconomic-geographic dimensions.

## Methods

### Data source

The present study used cross-sectional data from the 2015 Canadian Community Health Survey – Nutrition (CCHS-N)^(25)^. The 2015 CCHS-N dataset was selected because it contains the most recent, detailed and representative population data on the dietary intake of Canadians. The 2015 CCHS-N was a nationally representative survey that collected detailed information on dietary intake, along with other health-related information, for individuals living in Canada who were ≥ 1 year of age. Excluded from the sampling frame were people living on First Nations reserves, in Canada’s three northern Territories, in collective dwellings, in institutions and who were full-time members of the Canadian Forces (<3 % of the population).

Prior to data collection, Statistics Canada obtained informed consent from all participants in the 2015 CCHS-N^(25)^. The University of Waterloo’s Office of Research Ethics institutional review board deemed this research study exempt from ethical review, as per Article 2.2. of the Tri-Council Policy Statement 2 (2022)^(26)^.

### Data collection

Between January and December 2015, interviewers visited selected dwellings (based on a multi-stage sampling design) to collect basic household information and administered a general health questionnaire and a 24-h dietary recall^(25)^. Interviewers used the Automated Multiple-Pass Method, a computer-assisted five-step method, to guide respondents through recalling all foods and beverages consumed during the 24 h before the interview^(25)^. Using a set of standardised prompts and response options, interviewers obtained detailed information about the foods (including beverages) consumed by respondents^(25)^. For a subset of the sample, a second 24-h dietary recall was completed over the phone within 3–10 d of the first interview^(25)^; however, only the first-day recall was used to estimate mean population-level diet quality^(27)^.

From the adults ≥ 18 years of age in the dataset (*n* 14 275), we excluded individuals who were pregnant, breast-feeding or who reported no energy intake (2·2 %) and removed respondents with missing data (1·1 %). The final analytical sample for the primary models included data from 13 806 individuals.

### Measures

#### Canadian Marginalisation Index

**Figure 1.**
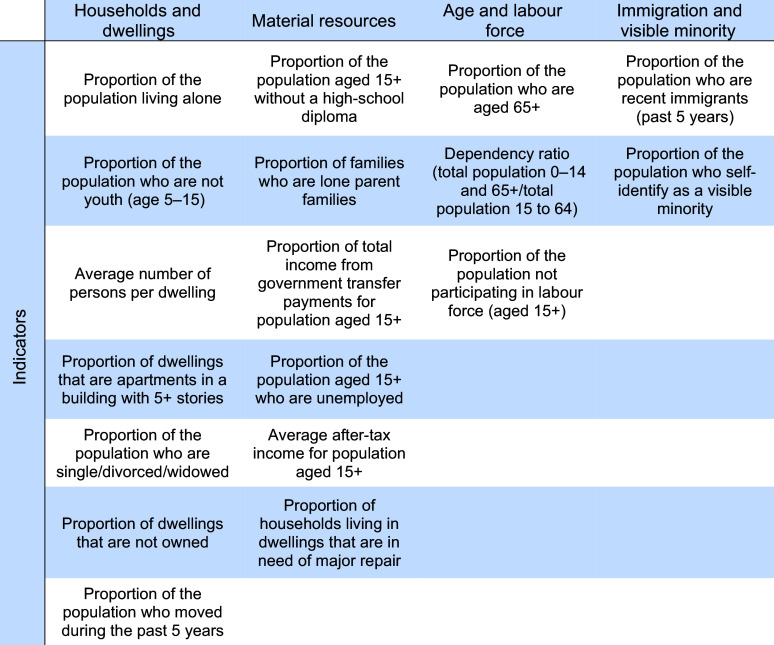
2016 CAN-Marg dimensions and their respective census indicators. *Notes*: Adapted from the Can-Marg 2016 User guide. Image description: Presented are the four CAN-Marg dimensions along with the 2016 census indicators that comprise each dimension. CAN-Marg, Canadian Marginalisation Index.

We used the Canadian Marginalisation Index (CAN-Marg) to assess neighbourhood deprivation in the Canadian context^(28)^. The CAN-Marg is a geography-based index comprising eighteen items associated with area-level deprivation and marginalisation^(28)^, classified into four dimensions: material resources, immigration and visible minority, age and labour force and households and dwellings (Figure 1)^(28)^. We used CAN-Marg values computed for 2016 Census Dissemination Areas (DA), which are small and stable geographic units averaging 400–700 people^(29)^, and the Postal Code Conversion File + version 6D^(30)^ to assign CCHS-N participants into DAs based on their residential postal codes. For each of the four dimension of neighbourhood deprivation, we assessed standardised factor scores, with lower scores indicating less neighbourhood deprivation and higher scores indicating more neighbourhood deprivation^(28)^.

#### Healthy Eating Index

The Healthy Eating Index-2015 (HEI-2015)^(6)^ was used to characterise diet quality. The HEI-2015 is based on the 2015–2020 Dietary Guidelines for Americans^(31)^, which broadly aligned with key dietary recommendations in Canada in 2015 (e.g. emphasis on vegetables, fruits, grains and protein foods, limits on Na, added sugar and saturated fats). The HEI-2015 has been extensively evaluated and applied to describe diet quality^(32)^. This index is made up of thirteen components, nine of which assess dietary *adequacy* (total fruits, whole fruits, total vegetables, greens and beans, whole grains, dairy, total protein foods, seafood and plant proteins and fatty acids) and four that assess dietary *moderation* (refined grains, sodium, added sugars and saturated fats)^(6)^. To convert food and nutrient intakes into the components necessary to calculate HEI-2015 scores, we used datasets that researchers had linked to food codes in the 2015 Canadian Nutrient File^(25,33)^, the 2015–2016 US Food and Nutrient Database for Dietary Studies^(34)^ and the 2015–2016 US Department of Agriculture’s Food Patterns Equivalents Database^(35)^. Person-level scores were calculated using the simple algorithm available from the National Cancer Institute^(36)^. Total HEI-2015 scores can range from zero to 100, with higher scores indicating better alignment with dietary guidance and therefore higher diet quality^(6)^.

#### Potential confounders

Individual-level characteristics, including age (continuous), sex/gender (male, female), Indigenous identity and race/ethnicity (Black, East Asian (Chinese, Japanese, Korean), Indigenous (First Nations, Métis and/or Inuk), Latin American, Middle Eastern (Arab, West Asian), South Asian, Southeast Asian (Southeast Asian, Filipino), White and another racial or ethnic group (individuals who selected ‘Other’ or multiple responses), immigration status (Canadian born, < 10 years since immigration, ≥ 10 years since immigration, temporary resident), marital status (married or common-law, not married or common law), employment status (employed, unemployed, not in the workforce) and smoking (not at all, occasionally and daily) were included in all models because of their established relationships with neighbourhood deprivation and diet quality^(37,38)^. All models were also adjusted for household and area-level factors associated with neighbourhood deprivation and diet quality, including household composition (unattached individuals living alone or with others, attached individuals living with a partner and children, single parents living with children, living with parents and another household composition), urbanicity (rural, urban)^(39)^ and province.

### Statistical analyses

#### Primary models

All analyses were conducted using SAS Enterprise^(40)^. Sampling weights and replicate bootstrap weights provided by Statistics Canada were applied to all analyses to account for the complex sampling design. Descriptive analyses were conducted to characterise the sample. Weighted linear regression was used to examine associations between neighbourhood deprivation and diet quality, adjusting for potentially confounding individual, household and area characteristics. We used fixed-effects rather than mixed-effects models since the number of individuals in each DA was fewer than the recommended thirty units at each level of a multi-level analysis^(41)^. Since CAN-Marg dimensions were not all correlated with HEI-2015 scores in the same direction^(28)^, we did not use CAN-Marg summary scores in our models.

For the primary analyses, we first assessed the main effects of each of the four CAN-Marg dimensions in relation to HEI-2015 scores. In this paper, ‘effects’ refer to statistical associations, not causal relationships. Second, given that different dimensions of neighbourhood deprivation may jointly influence diet quality, all possible two-way interactions among CAN-Marg dimensions were assessed in relation to HEI-2015 scores.

#### Secondary models

We developed models that adjusted for respondent highest educational attainment (less than a high school diploma, high school diploma, some post-secondary education, a bachelor’s degree or above), annual household income (very low, low, medium, high and very high) and household food insecurity status (food secure, marginally food insecure, moderately food insecure and severely food insecure) to examine the impact of including variables that could potentially mediate associations between neighbourhood deprivation and diet quality. Our annual household income measure was computed relative to the national distribution, based on deciles reflecting the ratio of total annual household income adjusted for the low-income cut-off by community and family size. Severity of household food insecurity was assessed using Health Canada’s validated Household Food Security Survey Module and scoring method^(42)^.

#### Sensitivity models

To examine the potential effects of dietary misreporting on associations between neighbourhood deprivation and diet quality, we developed models that adjusted for an indicator of dietary intake misreporting – the ratio of total energy intake to total energy expenditure, assuming a low physical activity level^(43)^.

## Results

### Sample characteristics

Among the weighted analytic sample, the mean age was 48·9 years and approximately equal proportions of individuals identified as male and female (Table 1). The highest proportions of individuals identified as White (73·6 %), were Canadian born (70·5 %), married or common-law (63·3 %), had some post-secondary school education (33·5 %) and did not smoke (81·4 %). The most common household composition was living with a partner and children (28·5 %). A majority of individuals lived in food-secure households (88·3 %), most lived in urban areas (82·7 %) and the highest proportion lived in the province of Ontario (38·8 %). The standardised factor scores for each CAN-Marg dimension had a mean close to zero, while the HEI-2015 scores averaged 57·9/100·0.

**Table 1.** Weighted characteristics of adults who participated in the 2015 Canadian Community Health Survey – Nutrition (*n* 13 806) Table 1 long description.

Variable	%^*^	se
Mean age, years	48·9	0·1
Sex/gender		
Male	49·9	0·1
Female	50·1	0·1
Indigenous identity and race/ethnicity^†^		
Black	3·4	0·4
East Asian	5·3	0·4
Indigenous (First Nation, Inuk/Inuit, Métis)	2·7	0·2
Latin American	1·3	0·2
Middle Eastern	2·4	0·3
South Asian	4·5	0·4
Southeast Asian	3·5	0·4
White	73·6	0·9
Another racial/ethnic group	3·4	0·4
Immigration status		
Temporary resident	2·1	0·3
Recent immigrant	6·9	0·5
Longer-term immigrant	20·6	0·8
Canadian born	70·5	0·9
Marital status		
Married or common law	63·3	0·8
Not married or common law	36·7	0·8
Employment status		
Employed	63·9	0·7
Unemployed	28·6	0·7
Not in the labour force	7·5	0·2
Smoking		
Daily smoker	14·0	0·6
Occasional or former smoker	4·6	0·3
Not at all	81·4	0·7
Household composition		
Unattached living alone with others	25·0	0·8
Living with a partner	27·8	0·7
Living with a partner and children	28·5	0·8
Single parent living with children	4·5	0·4
Living with parents	8·3	0·6
Another household composition	5·9	0·4
Urbanicity		
Urban	82·7	0·8
Rural	17·3	0·8
Province		
Newfoundland and Labrador	1·5	0·0
Prince Edward Island	0·4	0·0
Nova Scotia	2·7	0·0
New Brunswick	2·1	0·0
Quebec	23·7	0·1
Ontario	38·8	0·1
Manitoba	3·3	0·0
Saskatchewan	2·9	0·0
Alberta	11·3	0·1
British Colombia	13·2	0·1
Respondent educational attainment^‡^		
Less than a high school diploma or equivalent	11·8	0·5
High school diploma or equivalent	26·7	0·8
Some post-secondary school education	33·5	0·8
Bachelor’s degree or above	27·4	0·9
Not stated/don’t know/refusal	0·6	0·1
Annual household income		
Very low	19·8	0·7
Low	20·1	0·7
Middle	19·4	0·6
High	19·5	0·7
Very high	21·2	0·8
Household food insecurity status		
Severely food insecure	2·4	0·3
Moderately food insecure	5·4	0·3
Mildly food insecure	3·6	0·3
Food secure	88·3	0·6
Not stated/don’t know/refusal	0·2	0·1
Mean households and dwellings factor scores	0·0	0·0
Mean material resources factor scores	−0·1	0·0
Mean age and labour force factor scores	−0·1	0·0
Mean immigration and visible minority factor score	0·2	0·0
Total HEI-2015	57·9	0·2

HEI-2015, Healthy Eating Index-2015; %, percentage.

* In accordance with Statistics Canada’s confidentiality policies, all estimates incorporate sampling weights and replicate bootstrap weights provided by Statistics Canada. Standard errors represent the variability in the percentage across bootstrapped samples.

† Another racial/ethnic group includes individuals who selected ‘Other’ or multiple response options that did not fall exclusively within our race/ethnicity categories.

‡ Some post-secondary education includes collège d’enseignement général et professionnel, trade school, college, non-university certificates/diplomas, university certificates/diplomas below the bachelor level. Bachelor’s degree or above includes bachelor’s degree and university certificates/diplomas/degrees above the bachelor’s level.

### Primary models

#### Main effects models

After adjusting for confounding variables, individuals living in neighbourhoods with lower access to material resources (*β* = –0·955, 95 % CI = –1·438, –0·471; Table 2) and with a lower proportion of recent immigrants and visible minorities (*β* = 0·768, 95 % CI = 0·268, 1·268) had lower average HEI-2015 scores. The households and dwellings (*β* = –0·140, 95 % CI = –0·614, 0·333) and age and labour force dimensions were not significantly associated with HEI-2015 scores (*β* = –0·116, 95 % CI = –0·615, 0·383).

**Table 2.** Primary models testing the main effects and two-way interactions between each CAN-Marg dimension in relation to HEI-2015 scores (*n* 13 806) Table 2 long description.

Dimensions	*β* *	95 % CI	*P* *
Main effects			
Households and dwellings	−0·140	–0·614, 0·333	0·561
Material resources	**−0·955**	**–1·438, –0·471**	**<0·001**
Age and labour force	−0·116	–0·615, 0·383	0·649
Immigration and visible minority	0·768	0·268, 1·268	**0·003**
Two-way interactions			
Households and dwellings × material resources	0·231	–0·166, 0·628	0·254
Material resources × age and labour force	**−0·643**	**–1·052, –0·234**	**0·002**
Age and labour force × immigration and visible minority	0·501	–0·032, 1·034	0·065
Immigration and visible minority × households and dwellings	0·128	–0·293, 0·549	0·550
Households and dwellings × age and labour force	−0·087	–0·447, 0·273	0·635
Material resources × immigration and visible minority	**0·521**	**0·174, 0·868**	**0·003**

CAN-Marg, Canadian Marginalisation Index; HEI-2015, Healthy Eating Index-2015; *β*, beta estimate.

* In accordance with Statistics Canada’s confidentiality policies, all estimates incorporate sampling weights and replicate bootstrap weights provided by Statistics Canada. Bolded estimates are significant at *α* = 0·05. Estimates adjusted for age, sex/gender, Indigenous identity and race/ethnicity, immigration status, marital status, smoking, household composition, urbanicity and province.

#### Interaction models

After adjusting for confounding variables, the interaction between the material resources dimension and the age and labour force dimension was significant (*β* = –0·643, 95 % CI = –1·052, –0·234), indicating that the association between living in a neighbourhood with limited material resources and lower HEI-2015 scores was stronger for those living in neighbourhoods with a lower proportion of people in the labour force. The interaction between the material resources and immigration and visible minority dimensions was significant (*β* = 0·521, 95 % CI = 0·174, 0·868), indicating that association between living in a neighbourhood with less material resources and lower HEI-2015 scores was stronger for those living in neighbourhoods with a smaller proportion of recent immigrants and visible minorities. No other interactions were significantly associated with HEI-2015 scores.

#### Secondary models

After adjusting for respondent educational attainment, annual household income and household food insecurity status, the immigration and visible minority dimension (*β* = 0·774, 95 % CI = 0·270, 1·277) remained significantly associated with HEI-2015 scores; however, the material resources dimension (*β* = −0·431, 95 % CI = –0·935, 0·072) was no longer significant. (Table 3). The interaction between the material resources and age and labour force dimensions (*β* = –0·562, 95 % CI = –0·962, −0·162) and the interaction between the material resources and immigration and visible minority dimensions (*β* = 0·376, 95 % CI = 0·012, 0·741) remained significant.

**Table 3.** Secondary models testing the main effects and two-way interactions between each CAN-Marg dimension in relation to HEI-2015 scores (*n* 13 698) Table 3 long description.

Dimensions	*β* *	95 % CI	*P* *
Main effects			
Households and dwellings	−0·084	–0·568, 0·400	0·734
Material resources	−0·431	–0·935, 0·072	0·093
Age and labour force	−0·081	–0·557, 0·394	0·737
Immigration and visible minority	**0·774**	**0·270, 1·277**	**0·003**
Two-way interactions			
Households and dwellings × material resources	0·124	–0·287, 0·535	0·552
Material resources × age and labour force	**−0·562**	**–0·962, –0·162**	**0·006**
Age and labour force × immigration and visible minority	0·392	–0·153, 0·937	0·277
Immigration and visible minority × households and dwellings	0·073	–0·368, 0·514	0·746
Households and dwellings × age and labour force	−0·061	–0·416, 0·294	0·736
Material resources × immigration and visible minority	**0·376**	**0·012, 0·741**	**0·043**

CAN-Marg, Canadian Marginalisation Index; HEI-2015, Healthy Eating Index-2015; *β*, beta estimate.

* In accordance with Statistics Canada’s confidentiality policies, all estimates incorporate sampling weights and replicate bootstrap weights provided by Statistics Canada. Bolded estimates are significant at *α* = 0·05. Estimates adjusted for age, sex/gender, Indigenous identity and race/ethnicity, immigration status, marital status, smoking, household composition, urbanicity, province, educational attainment, annual household income and household food insecurity status.

#### Sensitivity models

The results of the sensitivity analyses that adjusted for total energy intake to energy expenditure ratio were consistent with our primary models (Table 4).

**Table 4.** Sensitivity models testing the main effects and two-way interactions between each CAN-Marg dimension in relation to HEI-2015 scores (*n* 13 126) Table 4 long description.

Dimensions	*β* *	95 % CI	*P* *
Main effects			
Households and dwellings	−0·096	–0·575, 0·382	0·693
Material resources	**−0·996**	**–1·495, –0·496**	**< 0·001**
Age and labour force	−0·185	–0·692, 0·323	0·476
Immigration and visible minority	**0·679**	**0·163, 1·195**	**0·010**
Two-way interactions			
Households and dwellings × material resources	0·251	–0·154, 0·657	0·224
Material resources × age and labour force	**−0·612**	**–1·038, –0·186**	**0·005**
Age and labour force × immigration and visible minority	0·488	–0·064, 1·040	0·083
Immigration and visible minority × households and dwellings	0·136	–0·296, 0·569	0·536
Household and dwellings × age and labour force	0·121	–0·489, 0·248	0·521
Material resources × immigration and visible minority	**0·499**	**0·132, 0·867**	**0·008**

CAN-Marg, Canadian Marginalisation Index; HEI-2015, Healthy Eating Index-2015; *β*, beta estimate.

* In accordance with Statistics Canada’s confidentiality policies, all estimates incorporate sampling weights and replicate bootstrap weights provided by Statistics Canada. Bolded estimates are significant at *α* = 0·05. Estimates adjusted for age, sex/gender, Indigenous identity and race/ethnicity, immigration status, marital status, smoking, household composition, urbanicity, province and the total energy intake to energy expenditure ratio.

## Discussion

Given limited knowledge about interactions between dimensions of neighbourhood deprivation and diet quality, we examined whether and to what extent four dimensions of neighbourhood deprivation were independently and jointly associated with diet quality among a nationally representative sample of adults in Canada. We found that living in a neighbourhood with less material resources was associated with lower diet quality, while living in a neighbourhood with a higher proportion of recent immigrants and visible minorities was associated with higher diet quality. Two-way interactions between the material resources, immigration and visible minority and age and labour force dimensions revealed that some dimensions of neighbourhood deprivation were jointly associated with diet quality.

We found that two out of four dimensions of neighbourhood deprivation were independently associated with diet quality. First, individuals living in neighbourhoods with less material resources were more likely to have lower diet quality. This finding is supported by a study by Murphy *et al.* (2022), which found that individuals living in neighbourhoods with very high material deprivation were less likely to meet fruit and vegetable consumption recommendations compared with those living in areas with very low material deprivation^(19)^. Although neighbourhood factors may impact the accessibility and affordability of healthier foods in more materially deprived neighbourhoods^(44,45)^, residents’ educational attainment, annual household income and food insecurity status may be confounders of the association between the neighbourhood material resource dimension and diet quality, as this main effect was no longer significant, after adjusting for these variables.

Second, living in a neighbourhood with a higher proportion of recent immigrants and visible minorities was associated with higher diet quality, independent of respondents’ race/ethnicity and immigration status and other individual- and household-level characteristics. It is therefore important to consider features of the social and/or built environments that could explain these associations. For example, it is possible that areas with higher concentrations of recent immigrants and visible minorities have greater demand for and therefore higher access to food retailers that sell culturally relevant foods from regions where many immigrants and visible minorities have ancestry (e.g. East Asia, South Asia and Middle East). The diets from these regions often emphasise whole, minimally processed and nutrient-dense foods (e.g. vegetables, fruit, pulses, legumes, fish and seafood), in contrast to dominant Western dietary patterns, which are often higher in energy-dense, nutrient-poor and ultra-processed foods^(46)^.

The significant interaction between the material resources dimension and the immigration and visible minority dimension indicates that there was a stronger association between living in a neighbourhood with less material resources and lower diet quality for individuals who also lived in neighbourhoods with a smaller proportion of recent immigrants and visible minorities. Recent immigrants and visible minorities may be more likely to reside in neighbourhoods with more material deprivation than non-immigrants/visible minorities^(47)^. This suggests that some characteristics of the areas where many recent immigrants and visible minorities live, such as more healthful retail food environments (as described above), may buffer the negative effects of neighbourhood material deprivation on diet quality. Therefore, inequities in diet quality based on neighbourhood deprivation may be amenable to changes to the built or food environment, for example, by increasing access to diverse food retailers that are more prevalent in areas with higher concentrations of immigrants and visible minorities^(48)^.

Although the age and labour force dimension was not independently associated with diet quality, there was a significant interaction between the material resources dimension and the age and labour force dimension. This significant interaction suggests that living in a neighbourhood with a smaller proportion of non-working individuals is not broadly associated with diet quality, but when combined with higher neighbourhood material deprivation, is associated with poorer diet quality. This finding extends previous research suggesting that area-level unemployment may be most strongly associated with poorer diet quality in neighbourhoods with higher material deprivation^(49,50)^. It is important to note, however, that the age and labour force dimension is based not only on the proportion of non-working individuals but also on the ratio of individuals aged < 15 and 65+ to those aged 15–65. Given the paucity of research on how neighbourhood age and employment composition are associated with diet quality, further research is warranted to understand these associations.

### Strengths and limitations

To our knowledge, this study was the first to assess joint associations between multiple dimensions of neighbourhood deprivation and diet quality. By leveraging data from the 2015 CCHS-N, we were able to assess these associations among a nationally representative sample of adults in Canada. We expanded on existing literature, which has primarily examined how neighbourhood deprivation relates to intake of fruits, vegetables and fast-food by evaluating overall diet quality^(22)^. By using intersectionality as a context-informed analytic tool to investigate differences in diet quality across the intersections of multiple socio-economic-geographic contexts, we were able to identify joint associations between dimensions of neighbourhood deprivation and diet quality that had not been previously identified.

There are also several limitations to consider. This study uses the most recent nationally representative data available; however, the data were collected more than 10 years ago. Although neighbourhood environments and dietary patterns have evolved, social, economic and environmental factors on diet quality tend to change gradually and remain relevant over time. Additionally, due to the cross-sectional and observational nature of the data, we cannot make causal inferences about the temporal relationships between variables. Moreover, since we assessed neighbourhood deprivation based on respondents’ residential postal codes, our measures of neighbourhood deprivation capture only *residential* neighbourhood deprivation and not other neighbourhoods where people may work, study and play.

With respect to characterising diet quality, we relied on self-reported 24-h dietary recall data, which may contain errors due to misreporting^(51)^. To assess the potential impact of misreporting on our estimates, we conducted sensitivity analyses that adjusted for the total energy intake to energy expenditure ratio as an indicator of dietary misreporting and found consistent results. Lastly, because we relied on a single dietary recall, we were not able to account for within-person variations in dietary intake^(52)^; however, a single recall captures mean diet quality at the population level^(27)^.

### Conclusion

In conclusion, we found that dimensions of neighbourhood deprivation were independently and jointly associated with diet quality among a nationally representative sample of adults in Canada. Since some dimensions of neighbourhood deprivation were jointly associated with diet quality, our research underscores the importance of using intersectionality as a context-informed tool to investigate dietary inequities at the neighbourhood level. Additionally, although individuals living in neighbourhoods with less material resources were more likely to have lower diet quality, significant interactions with the immigration and visible minority dimension, as well as the age and labour force dimension, suggest that inequities in diet quality based on neighbourhood material deprivation are not ubiquitous. Therefore, inequities in diet quality based on neighbourhood deprivation may be amenable to changes to the built or food environment, such as increasing access to nutritious and affordable foods.

## Table 1. Long description

A table showing characteristics of adults in the 2015 Canadian Community Health Survey. The table has 40 rows and 4 columns. The columns are labeled Variable, %, and SE. The rows list various characteristics and their corresponding percentages and standard errors. Row 1: Mean age, years, 48.9, 0.1. Row 2: Sex/gender, Male, 49.9, 0.1. Row 3: Sex/gender, Female, 50.1, 0.1. Row 4: Indigenous identity and race/ethnicity, Black, 3.4, 0.1. Row 5: Indigenous identity and race/ethnicity, East Asian, 5.3, 0.2. Row 6: Indigenous identity and race/ethnicity, Indigenous (First Nation, Inuit, Metis), 2.7, 0.2. Row 7: Indigenous identity and race/ethnicity, Latin American, 1.3, 0.1. Row 8: Indigenous identity and race/ethnicity, Middle Eastern, 2.4, 0.1. Row 9: Indigenous identity and race/ethnicity, South Asian, 4.5, 0.2. Row 10: Indigenous identity and race/ethnicity, Southeast Asian, 3.5, 0.2. Row 11: Indigenous identity and race/ethnicity, White, 73.6, 0.4. Row 12: Indigenous identity and race/ethnicity, Another racial/ethnic group, 3.4, 0.2. Row 13: Immigration status, Temporary resident, 2.1, 0.2. Row 14: Immigration status, Recent immigrant, 6.9, 0.3. Row 15: Immigration status, Longer-term immigrant, 20.6, 0.4. Row 16: Immigration status, Canadian born, 70.5, 0.4. Row 17: Marital status, Married or common law, 63.3, 0.4. Row 18: Marital status, Not married or common law, 36.7, 0.4. Row 19: Employment status, Employed, 63.9, 0.4. Row 20: Employment status, Unemployed, 28.6, 0.4. Row 21: Employment status, Lost in the labour force, 7.5, 0.2. Row 22: Smoking, Daily smoker, 14.0, 0.3. Row 23: Smoking, Occasional or former smoker, 46.0, 0.4. Row 24: Smoking, Not at all, 81.4, 0.3. Row 25: Household composition, Unattached living alone or others, 25.0, 0.3. Row 26: Household composition, Living with a partner, 27.8, 0.3. Row 27: Household composition, Living with a partner and children, 28.5, 0.3. Row 28: Household composition, Single parent living with children, 8.3, 0.2. Row 29: Household composition, Living with parents, 9.5, 0.2. Row 30: Household composition, Another household composition, 5.9, 0.2. Row 31: Urban/rural, Urban, 82.7, 0.3. Row 32: Urban/rural, Rural, 17.3, 0.3. Row 33: Province, Newfoundland and Labrador, 1.7, 0.1. Row 34: Province, Prince Edward Island, 0.4, 0.0. Row 35: Province, Nova Scotia, 2.7, 0.1. Row 36: Province, New Brunswick, 2.1, 0.1. Row 37: Province, Quebec, 23.7, 0.3. Row 38: Province, Ontario, 38.8, 0.4. Row 39: Province, Manitoba, 3.3, 0.1. Row 40: Province, Saskatchewan, 2.9, 0.1. Row 41: Province, Alberta, 11.3, 0.2. Row 42: Province, British Columbia, 13.2, 0.2. Row 43: Respondent educational attainment, Less than a high school diploma or equivalent, 11.8, 0.3. Row 44: Respondent educational attainment, High school diploma or equivalent, 26.7, 0.3. Row 45: Respondent educational attainment, Some post-secondary school education, 33.5, 0.3. Row 46: Respondent educational attainment, Bachelor's degree or above, 27.4, 0.3. Row 47: Respondent educational attainment, Not stated/don't know/refusal, 0.6, 0.0. Row 48: Annual household income, Very low, 19.8, 0.4. Row 49: Annual household income, Low, 20.1, 0.4. Row 50: Annual household income, Middle, 19.4, 0.4. Row 51: Annual household income, High, 19.5, 0.4. Row 52: Annual household income, Very high, 21.2, 0.4. Row 53: Household food insecurity status, Severely food insecure, 2.4, 0.1. Row 54: Household food insecurity status, Moderately food insecure, 5.4, 0.2. Row 55: Household food insecurity status, Mildly food insecure, 3.6, 0.1. Row 56: Household food insecurity status, Food secure, 88.3, 0.3. Row 57: Household food insecurity status, Not stated/don't know/refusal, 0.2, 0.0. Row 58: Mean household and dwelling factor scores, Mean household and dwelling factor scores, 0.0, 0.0. Row 59: Mean age and labour force factor scores, Mean age and labour force factor scores, -0.1, 0.0. Row 60: Mean immigration and visible minority factor score, Mean immigration and visible minority factor score, 0.2, 0.0. Row 61: Mean household composition factor score, Mean household composition factor score, 0.0, 0.0. Row 62: Total HEI-2015, Total HEI-2015, 57.9, 0.2.

Navigate back to Table 1..

## Table 2. Long description

A table titled Primary models testing the main effects and two-way interactions between each CAN-Marg dimension in relation to HEI-2015 scores (n 13 806). The table has 11 rows and 4 columns. The columns are labeled Dimensions, beta, 95% CI, and P value. The table is divided into two sections: Main effects and Two-way interactions. Row 1: Households and dwellings, beta -0.140, 95% CI -0.614, 0.333, P value 0.561. Row 2: Material resources, beta -0.955, 95% CI -1.438, -0.471, P value < 0.001. Row 3: Age and labour force, beta -0.116, 95% CI -0.615, 0.383, P value 0.649. Row 4: Immigration and visible minority, beta 0.768, 95% CI 0.268, 1.268, P value 0.003. Row 5: Households and dwellings x material resources, beta 0.231, 95% CI -0.166, 0.628, P value 0.254. Row 6: Material resources x age and labour force, beta -0.643, 95% CI -1.052, -0.234, P value 0.002. Row 7: Age and labour force x immigration and visible minority, beta 0.501, 95% CI -0.032, 1.034, P value 0.065. Row 8: Immigration and visible minority x households and dwellings, beta 0.128, 95% CI -0.293, 0.549, P value 0.550. Row 9: Households and dwellings x age and labour force, beta -0.087, 95% CI -0.447, 0.273, P value 0.635. Row 10: Material resources x immigration and visible minority, beta 0.521, 95% CI 0.174, 0.868, P value 0.003.

Navigate back to Table 2..

## Table 3. Long description

A table with three columns and eleven rows. The columns are labeled Dimensions, beta, 95 percent CI, and P value. The table is divided into two sections: Main effects and Two-way interactions. The Main effects section includes rows for Households and dwellings, Material resources, Age and labour force, and Immigration and visible minority. The Two-way interactions section includes rows for Households and dwellings x material resources, Material resources x age and labour force, Age and labour force x immigration and visible minority, Immigration and visible minority x households and dwellings, Households and dwellings x age and labour force, and Material resources x immigration and visible minority. Each row contains values for beta, 95 percent CI, and P value. Notable trends include significant associations for Immigration and visible minority, Material resources x age and labour force, and Material resources x immigration and visible minority.

Navigate back to Table 3..

## Table 4. Long description

A table showing sensitivity models for main effects and interactions between CAN-Marg dimensions and HEI-2015 scores. The table has 11 rows and 4 columns. The columns are labeled Dimensions, Beta, 95% CI, and P value. The rows are grouped into Main effects and Two-way interactions. The table includes the following data: Row 1: Households and dwellings, -0.096, -0.575, 0.382, 0.693. Row 2: Material resources, -0.996, -1.495, -0.496, < 0.001. Row 3: Age and labour force, -0.185, -0.692, 0.323, 0.476. Row 4: Immigration and visible minority, 0.679, 0.163, 1.195, 0.010. Row 5: Households and dwellings x material resources, 0.251, -0.154, 0.657, 0.224. Row 6: Material resources x age and labour force, -0.612, -1.038, -0.186, 0.005. Row 7: Age and labour force x immigration and visible minority, 0.488, -0.064, 1.040, 0.083. Row 8: Immigration and visible minority x households and dwellings, 0.136, -0.296, 0.569, 0.536. Row 9: Household and dwellings x age and labour force, 0.121, -0.489, 0.248, 0.521. Row 10: Material resources x immigration and visible minority, 0.499, 0.132, 0.867, 0.008.

Navigate back to Table 4..
